# Supplementary material for: A Critical View of the Application of the APEX Software (Aqueous Photochemistry of Environmentally-Occurring Xenobiotics) to Predict Photoreaction Kinetics in Surface Freshwaters
Source: Molecules. 2019 Dec 18;25(1):9. doi: 10.3390/molecules25010009 (PMC7017383; doi:10.3390/molecules25010009)
Supplement: Supplementary file 1 [file molecules-25-00009-s001.zip › 3.2.4_gcc-4.4.0.zip!/3.2.4_gcc-4.4.0/3.2.4_gcc-4.4.0/doc/octave/html/interpreter/Standalone-Programs.html]

Standalone Programs - Untitled


Previous: Mex-Files,
Up: Dynamically Linked Functions


---

### A.3 Standalone Programs

The libraries Octave itself uses, can be utilized in standalone
applications. These applications then have access, for example, to the
array and matrix classes as well as to all the Octave algorithms. The
following C++ program, uses class Matrix from liboctave.a or
liboctave.so.

```
```
     #include <iostream>
     #include <octave/oct.h>
     
     int
     main (void)
     {
       std::cout << "Hello Octave world!\n";
       int n = 2;
       Matrix a_matrix = Matrix (n, n);
       for (octave_idx_type i = 0; i < n; i++)
         {
           for (octave_idx_type j = 0; j < n; j++)
             {
               a_matrix (i, j) = (i + 1) * 10 + (j + 1);
             }
         }
       std::cout << a_matrix;
       return 0;
     }
```
```

mkoctfile can then be used to build a standalone application with a
command like

```
     $ mkoctfile --link-stand-alone standalone.cc -o standalone
     $ ./standalone
     Hello Octave world!
       11 12
       21 22
     $
```

Note that the application `hello` will be dynamically linked
against the octave libraries and any octave support libraries. The above
allows the Octave math libraries to be used by an application. It does
not however allow the script files, oct-files or builtin functions of
Octave to be used by the application. To do that the Octave interpreter
needs to be initialized first. An example of how to do this can then be
seen in the code

```
```
     #include <iostream>
     #include <octave/oct.h>
     #include <octave/octave.h>
     #include <octave/parse.h>
     
     int
     main (void)
     {
       string_vector argv (2);
       argv(0) = "embedded";
       argv(1) = "-q";
     
       octave_main (2, argv.c_str_vec(), 1);
     
       octave_idx_type n = 2;
       Matrix a_matrix = Matrix (1, 2);
     
       std::cout << "GCD of [";
       for (octave_idx_type i = 0; i < n; i++)
         {
           a_matrix (i) = 5 * (i + 1); 
           if (i != 0)
     	std::cout << ", " << 5 * (i + 2);
           else
     	std::cout << 5 * (i + 2);
         }
       std::cout << "] is ";
     
       octave_value_list in = octave_value (a_matrix);
       octave_value_list out = feval ("gcd", in, 1);
     
       if (!error_state && out.length () > 0)
         {
           a_matrix = out(0).matrix_value ();
           if (a_matrix.numel () == 1)
     	std::cout << a_matrix(0) << "\n";
           else
     	std::cout << "invalid\n";
         }
       else
         std::cout << "invalid\n";
     
       return 0;
     }
```
```

which is compiled and run as before as a standalone application with

```
     $ mkoctfile --link-stand-alone embedded.cc -o embedded
     $ ./embedded
     GCD of [10, 15] is 5
     $
```
